# Carbonate determination in soils by mid-IR spectroscopy with regional and continental scale models

**DOI:** 10.1371/journal.pone.0210235

**Published:** 2019-02-21

**Authors:** Jonathan P. Comstock, Sonam R. Sherpa, Richard Ferguson, Scarlett Bailey, Jeffrey P. Beem-Miller, Feng Lin, Johannes Lehmann, David W. Wolfe

**Affiliations:** 1 Horticulture Section, School of Integrative Plant Science, Cornell University, Ithaca, New York, United States of America; 2 Kellogg Soil Survey Laboratory, Natural Resources Conservation Service, Lincoln, Nebraska, United States of America; 3 Soil and Crop Sciences Section, School of Integrative Plant Sciences, Cornell University, Ithaca, New York, United States of America; 4 Jiangsu Key Laboratory of Low Carbon Agriculture and GHGs Mitigation, Nanjing Agricultural University, Nanjing, China; 5 Atkinson Center for a Sustainable Future, Cornell University, Ithaca, New York, United States of America; The University of Sydney, AUSTRALIA

## Abstract

A Partial Least Squares (PLS) carbonate (CO_3_) prediction model was developed for soils throughout the contiguous United States using mid-infrared (MIR) spectroscopy. Excellent performance was achieved over an extensive geographic and chemical diversity of soils. A single model for all soil types performed very well with a root mean square error of prediction (RMSEP) of 12.6 g kg^-1^ and was further improved if Histosols were excluded (RMSEP 11.1 g kg^-1^). Exclusion of Histosols was particularly beneficial for accurate prediction of CO_3_ values when the national model was applied to an independent regional dataset. Little advantage was found in further narrowing the taxonomic breadth of the calibration dataset, but higher precision was obtained by running models for a restricted range of CO_3_. A model calibrated using only on the independent regional dataset, was unable to accurately predict CO_3_ content for the more chemically diverse national dataset. Ten absorbance peaks enabling CO_3_ prediction by mid-infrared (MIR) spectroscopy were identified and evaluated for individual and combined predictive power. A single-band model derived from an absorbance peak centered at 1796 cm^-^yielded the lowest RMSEP of 13.5 g kg^-1^ for carbonate prediction compared to other single-band models. This predictive power is attributed to the strength and sharpness of the peak, and an apparent minimal overlap with confounding co-occurring spectral features of other soil components. Drawing from the 10 identified bands, multiple combinations of 3 or 4 peaks were able to predict CO_3_ content as well as the full-spectrum national models. Soil CO_3_ is an excellent example of a soil parameter that can be predicted with great effectiveness and generality, and MIR models could replace direct laboratory measurement as a lower cost, high quality alternative.

## Introduction

In some arid and semi-arid regions, the soil inorganic carbon (SIC) in carbonates can be the dominant form of soil carbon. In moderately humid regions, SIC often complicates analytical determination of soil organic carbon (SOC), especially at greater depths [1]. While both SOC and SIC contribute important fractions of total soil carbon (TC) under various climatic regimes, SOC rather than SIC is of interest in relation to soil health, fertility, and carbon sequestration. SOC is considered the more dynamic component of TC, exchanging with atmospheric greenhouse gasses in a manner highly sensitive to land use and management practices, but ongoing studies suggest that SIC can also be variously manipulated to be a net source or sink of atmospheric CO_2_ under some circumstances [2–5]. The need for low-cost high-throughput measurement approaches for both SOC and SIC has grown tremendously in the last two decades to support studies of soil carbon dynamics requiring large amounts of data, real-time data-informed management practices, and inexpensive and rapid verification of soil carbon stocks to facilitate sequestration efforts and carbon markets [3,6–8].

A wide variety of direct methods have been developed for measuring CO_3_ contents in soils [9]. These include dry combustion at two temperatures for selectively removing first SOC and subsequently SIC [10] and methods based on acidifying soil samples and evaluating the release of CO_2_ from carbonates either gravimetrically [11], by titration, or manometrically [12–14].All of these laboratory methods require considerable time investments in sample preparation and measurement, and are consequently relatively low throughput and high-cost approaches. More recently interest has grown in spectroscopic methods of soil carbonate measurement using both visible and near- / mid- infrared spectral regions (VNIR and MIR, respectively) [6,15–17]. These approaches have shown great potential for high throughput, low cost per sample after initial investment in equipment, and the potential to evaluate numerous soil properties simultaneously. Nonetheless, questions remain regarding the limitations and generality of the spectroscopic prediction models.

Carbonate content has been one of the most tractable soil properties for MIR analysis [6,18,19]., but different forms of Carbonate are rarely distinguished in these studies. The most common form is Calcite (CaCO_3_) but many soils also have substantial amounts of Dolomite (CaMg(CO_3_)_2_), and several other rarer forms also occur in restricted cases. This is a potential source of error in calibration due to both spectral differences among CO_3_ minerals_,_ and differences in molecular weight per CO_3_ ion. The success in modeling CO_3_ with MIR chemometric models is due to the presence of several well-defined absorption peaks, and because soil carbonates, when present, can reach very high percentages of total soil mass with a correspondingly dominant influence on spectral properties. The numerous strong spectral features and large amounts of carbonate in some soils, however, can seriously confound the interpretation of other soil parameters. Strong carbonate peaks associated with fundamental vibrational states are present in the MIR region at 700, 880, and 1450 cm^-1^ [20]. Several additional bands, such as that at 3000–2900 cm^-1^ are due to overtones, and bands at 2600–2500 cm^-1^, and 1830–1760 cm^-1^ are due to combinations of fundamental vibrations [20–22].

Earlier evaluations assessing MIR measurement of soil CO_3_ were promising but often either very limited in scope or of more qualitative accuracy [23]. McCarty et al. [24], however, showed excellent MIR CO_3_ predictions (RMSEP = 10 g kg^-1^, bias 2.5 g kg^-1^) for a set of Alfisols and Mollisols from the central United States. A French national model achieved slightly less precision (RMSEP = 23 g kg^-1^) [15]. Our study seeks to clarify the trade-offs between high performance and generality across datasets with extensive chemical and geographic variability. Specifically, we will test:

**Hypothesis 1)** CO_3_ spectral characteristics are sufficiently strong and distinctive to allow a single predictive model to accurately predict CO_3_ content for all common soil types found in the contiguous United States provided the variation in soil types is well represented in a robust calibration dataset.
**Hypothesis 2)** Models derived from localized data or data with restricted soil diversity may achieve apparently lower error terms in internal validation, but lack generality outside the narrowly defined calibration limits.
**Hypothesis 3)** When calibrated against a sufficiently robust and extensive dataset, a broad inclusive model can match the accuracy and precision of other standard techniques for carbonate assessment despite extensive heterogeneity of soil background.

To address these questions, we report on a collaborative effort between the National Resources Conservation Service (NRCS) Kellogg Soil Survey Laboratory (KSSL) in Lincoln, Nebraska, and researchers at Cornell University in Ithaca, NY. The KSSL lab is the repository of a large soil archive with collections from the 1950s to the present. It currently houses approximately 245,000 soil samples and the archive is steadily growing. Approximately 55,000 samples from this archive have been scanned for MIR spectra as well as other measurements, and the total fraction scanned is steadily increasing. This spectral library and the associated information on soil properties allowed us to test these hypotheses on a national dataset with broad geographic distribution across the contiguous United States and representatives of the most important soil orders displayed on the map. Andisols, Spodisols andUltisols were not represented.

## Materials and methods

### Soil sample collection and preparation

**Fig 1:**
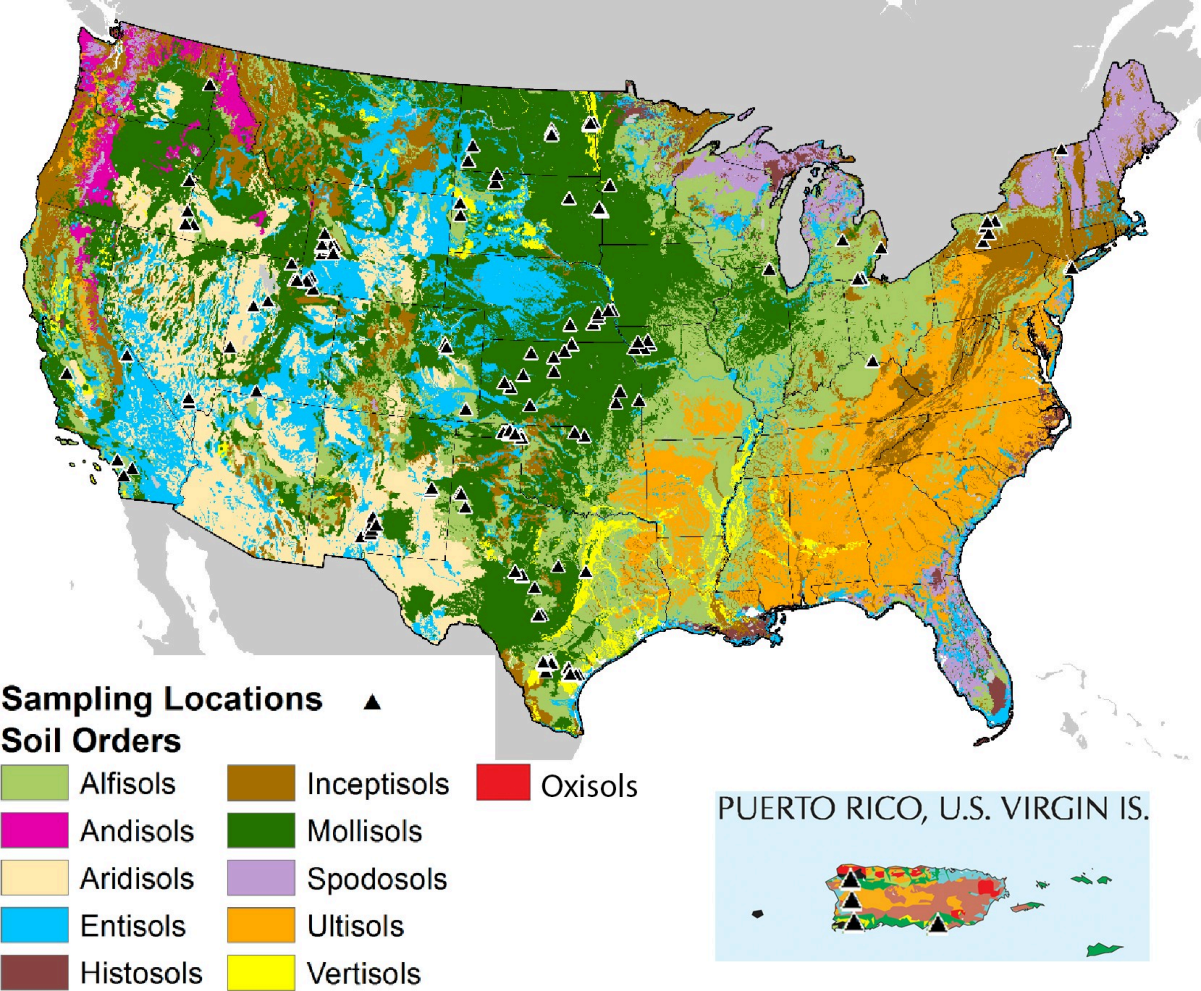
Dominant soil orders in the United States and locations of soil sample collection (triangles) included in the national CO_3_ model.

The national dataset used in this study includes 1268 samples containing CO_3_ from the KSSL archive and accessed through the Laboratory Information and Management System (LIMS database). These samples provide a broad survey of both geographic and chemical soil diversity (Table 1, Fig 1) across the contiguous United States and Puerto Rico. A second soil collection of 209 samples came from independent sampling by the Cornell team at three sites in New York State (Inceptisols and Alfisols) and two in Iowa (Mollisols) (Table 1).

**Table 1:** Geographic and taxonomic diversity of soils contributing to KSSL national CO_3_ model and the Cornell regional model.

Soil Order	number of soil samples			number of States		
	KSSL	Cornell	Total	KSSL	Cornell	Total
**Alfisols**	70	84	154	8	1	9
**Aridisols**	186	0	186	9	0	9
**Entisols**	32	0	32	1	0	1
**Histosols**	32	0	32	8	0	8
**Inceptisols**	70	96	166	13	1	14
**Mollisols**	497	29	526	5	1	5
**Vertisols**	95	0	95	11	0	11
**(Not recorded)**	286	0	286	11	0	11
**Total:**	1268	209	1477	29	2	30

The individual states contributing the most samples to the national dataset were North Dakota, Texas, Wyoming, New Mexico, South Dakota, and Kansas, with 403, 167, 116, 103, 98, and 60 samples, respectively. 18 samples came from Puerto Rico. New York and Iowa, the states sampled for development of the Cornell localized model, contributed only 25 and 1 sample, respectively. The Cornell dataset had Alfisols and Inceptisols sampled in New York and Mollisols from Iowa.

Samples for the national dataset were initially prepared at KSSL using method 1B1b2d1 [25] to achieve a < 2mm fraction of fine earth which was further ground to 80 mesh in a Fritsch Planetary mill with Syalon grinding vessel and balls. Samples very high in organic matter, such as Histosols, could not be ground in the planetary mill and were fine-ground using a cross-beater mill (Fritsch Pulverisette 16 mill with an 80 mesh screen). Samples at Cornell were prepared in similar fashion and finished with grinding in a Retsch MM200 ball mill with stainless steel grinding vessels and balls. The high levels of grinding needed to achieve sample homogeneity for the small subsamples in MIR measurement also have consequences for spectral characteristics [26]. Preliminary tests were run on common samples to ensure that equivalent spectra would be produced after sample prep at either location.

### Laboratory CO_3_ measurement

Results throughout this report are given as CaCO_3_ equivalents because the manometric method is calibrated using reagent gradeCaCO_3_ standards [13]. The measurement is based on measuring the CO_2_ released from carbonate reacting with 3M HCl, and is expected to be equally effective at measuring soil CO_3_ carbon released from the full range of carbonate soil minerals including common forms such as calcite, magnesite, and dolomite. Results are presented in units of CaCO_3_ equivalents, though actual weights and percent of total soil mass would differ depending on associated cations and crystal structures. Spectra of the common forms of soil carbonate can differ slightly, but generally show the same principle peaks in the MIR region [27,28].

CaCO_3_ equivalents were determined at both locations by pressure calcimeter method treating the <2mm soil fraction with 3M HCl in a closed vial. At KSSL this was method 4E1a1a1, pg 370 [25]. The method employed at Cornell University was similar in concept following the protocol of Sherrod et al. [13].

### MIR spectroscopy and CO_3_ prediction model development

All soil samples were analyzed by Diffuse Reflectance Infrared Fourier Transform (DRIFT)-MIR spectroscopy. Undiluted soil samples were laid out in aluminum 96 well microplates with four replicate wells of each soil sample resulting in four replicate spectra for each sample. Soil samples were scanned in a Bruker Vertex 70 FT-IR Spectrometer with HTS-XT (Bruker Optik GmbH, Germany). The HTS-XT is an external microplate module. Spectra were scanned from 600–4000 cm^−1^ with a resolution of 4 cm^−1^ and 32 scans/sample-well. Final spectra were expressed in absorbance units [log(1/Reflectance)]. Empty microplate wells with anodized aluminum bottoms were used as background.

### Influence of taxonomic coverage on model performance

**Fig 2:**
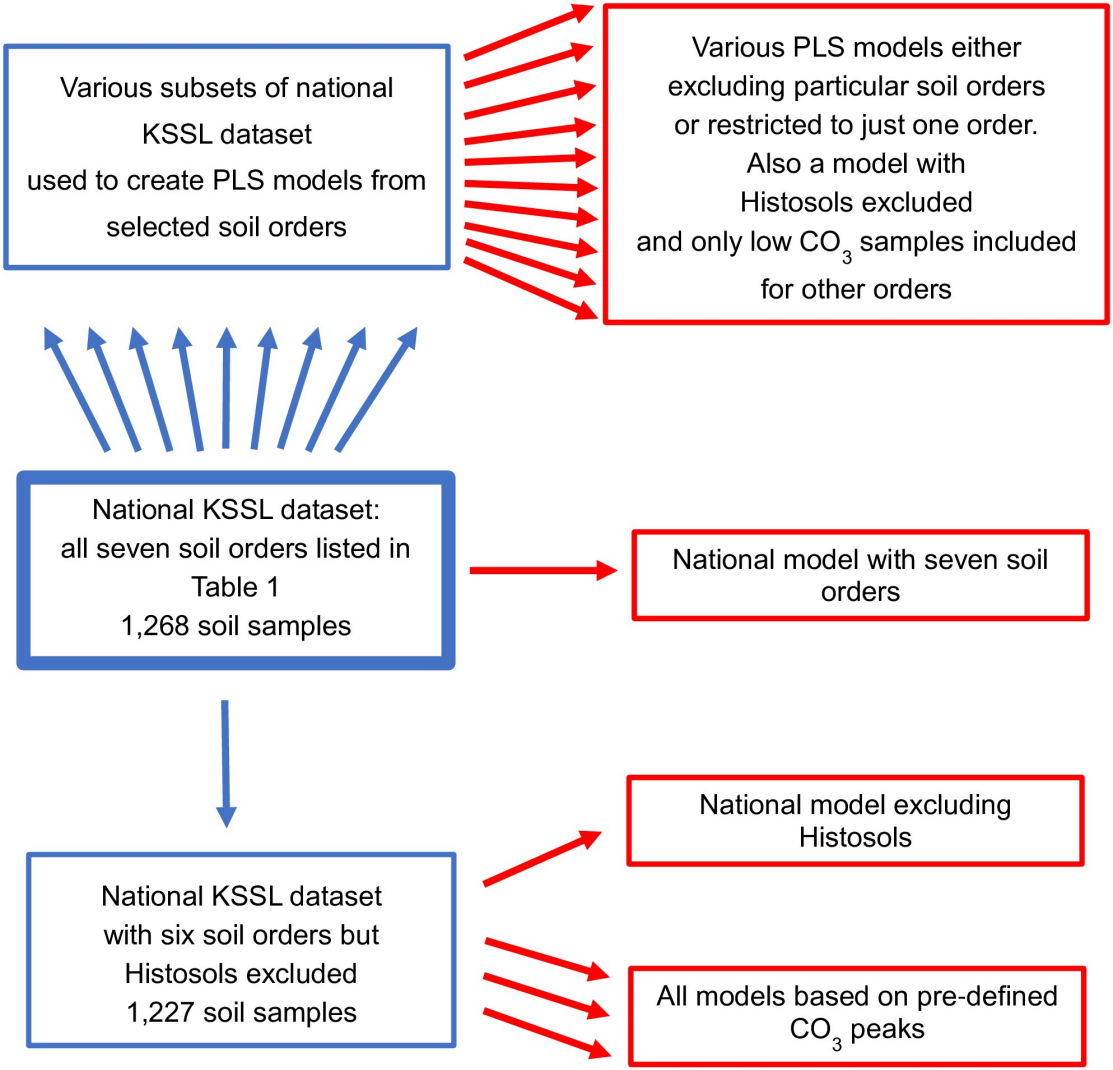
Flow chart of data subdivision and multiple model development starting with the full national dataset including Histosols. Blue boxes represent spectral and CO_3_ datasets, and red boxes the PLS models derived from them. Through preliminary principal components analysis (PCA) of the raw spectra using 10 principle components, 119 and 0 samples were excluded as redundant spectra from the KSSL national dataset and the smaller Cornell dataset, respectively. The remaining spectra in the national dataset were then divided, half for calibration and half for internal validation, using the Kennard-Stone algorithm to ensure equal distribution in the final PCA space. The smaller Cornell dataset was tested with leave-one-out cross validation (LOOCV). All chemometric models were developed using partial least squares regression (PLS) but with redundant spectra identified in PCA above excluded.

The national dataset was used to compare the performance of PLS models produced using the full dataset with an array of subsets focused on single soil orders or excluding various combinations of soil orders. The flow of spectral subset selection resulting in these various models is shown in Fig 2. The blue boxes both above and below the full national dataset in the flowchart are all subsets of various kinds. The national dataset with only Histosols excluded is recognized separately because of its importance in analyses of specific CO_3_ peaks.

PLS chemometric models were developed in part using the “optimization” function of the OPUS-QUANT2 software package (Bruker Optik GmbH, Germany). This automated routine considered 11 options for spectral preprocessing together with a predefined array of bands dividing up the spectrum from 4000 to 600 cm^-1^. This optimization routine did not employ a full factorial approach but started out testing each spectral pre-processing option using the whole spectrum and also leaving out various selected bands to determine which made important contributions. Spectral band choices were then refined during an iterative process, still including all preprocessing options in each iteration. Results of all tests conducted were then ranked based on root mean square error of the prediction (RMSEP) or the root mean square error of cross validation (RMSECV) as appropriate to determine the best combination of spectral preprocessing and spectral regions. Preprocessing options evaluated included: original spectra without data pre-processing, constant offset elimination, straight line subtraction, vector normalization, min max normalization, multiplicative scatter correction, first derivative, second derivative, first derivative with straight line subtraction, first derivative with vector normalization, and first derivative with multiplicative scatter correction. Prediction accuracy of selected MIR models was evaluated by the coefficient of determination (R^2^), the RMSEP or RMSECV, bias, standard error of prediction (SEP) and the residual prediction deviation (RPD) [29]. While most models were calibrated using a calibration and test set division during optimization and evaluated by RMSEP, the Cornell model and three single soil order models derived from the national dataset (Histosols, Aridisols and Entisols) were developed with the cross-validation leave one out technique due to reduced sample size and were evaluated with RMSECV.

Samples with CO_3_ contents above 70% were excluded because of reduced accuracy and insufficient sample size in that extreme range. This may have been related in part to spectral distortions of the undiluted samples as CO_3_ approaches the total composition of the sample [22]. Also excluded due to unresolved outlier status were samples with extremely high levels of soluble salts (e.g., salt playas). No data were excluded from the Cornell dataset and only a limited number of well-defined cases were excluded from the national model dataset.

### Reproducibility and accuracy of CO_3_ measurements

Laboratory standard soils included in KSSL measurement protocols permitted a comparison of reproducibility and accuracy between manometric and MIR techniques. KSSL laboratory standards 104 and 146 were included (routine quality assurance) during each run of the CO_3_ manometric assay, and 15 samples (60 wells in a 96 well plate) were also scanned by MIR for CO_3_ prediction using the KSSL (without Histosols) model. Another carbonate-containing KSSL laboratory standard, 101, is loaded on every 96 well plate during MIR analysis and has thus been scanned thousands of times. Standard 101 was subjected to 14 reps in a manometric analysis for comparison. Data are compared for mean values and standard deviations.

### Spectral regions associated with CO_3_ prediction by MIR

To evaluate the contribution of specific hypothesized carbonate peaks, a similar software-driven optimization process was employed only substituting the defined array of spectral bands for the default bands dividing up the entire spectrum. 16 samples were chosen with CO_3_ contents between 300 and 450 g kg^-1^ and equally representing all soil orders in the KSSL national dataset except Histosols. Examination of these spectra resulted in identification of 10 spectral bands consistently associated with high CO_3_. A set of ten prediction models was developed using the KSSL national dataset but limited to each of the listed spectral bands in turn. All models were constrained to use first derivative preprocessing, a maximum number of 15 PLS loading vectors, and the same division between test and calibration samples. This array of preliminary optimization results provided an assessment of the performance of individual carbonate peaks and also the combinations of combined peaks that were most effective. In all cases, whether using default spectral regions or the pre-assessed carbonate peaks, the best optimization results were run again with a full analysis graphically and statistically.

## Results & discussion

### Performance of national and regional scale CO_3_ prediction models

**Fig 3:**
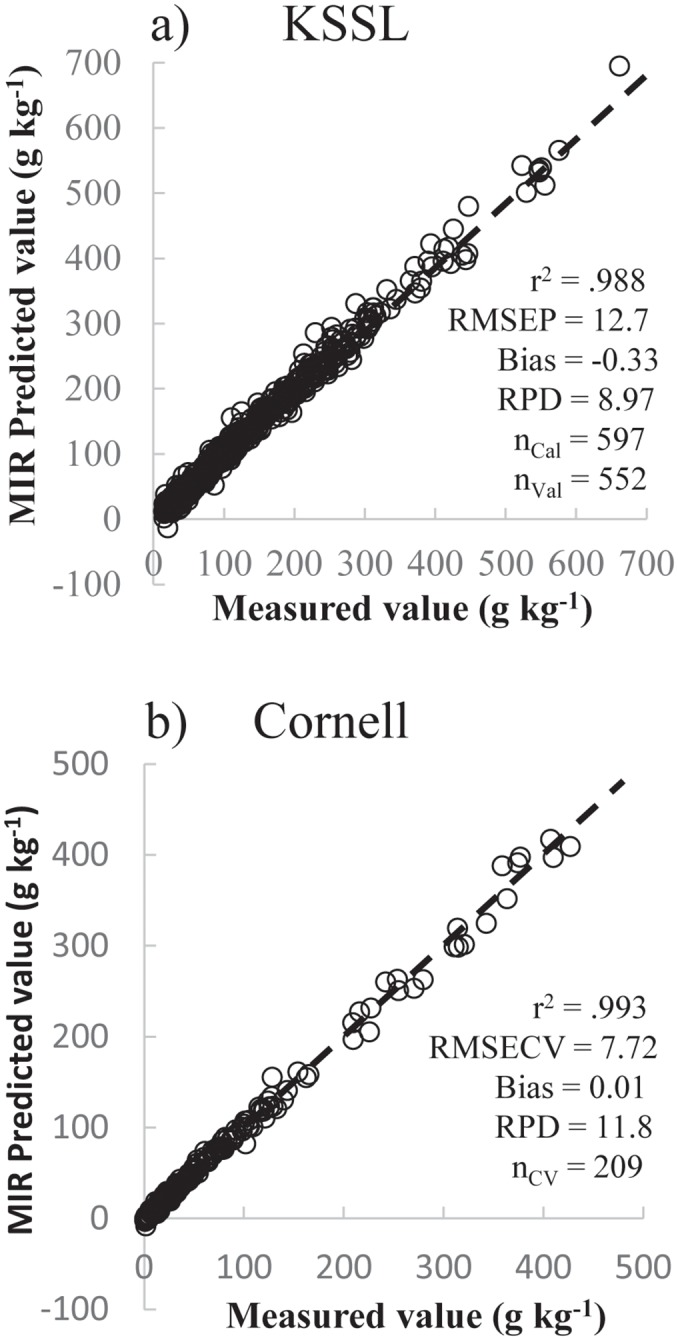
MIR modeled CaCO_3_ equivalent as percent of soil weight verses manometrically measured CaCO_3_ contents. **A**) National model based on 1268 samples from the KSSL archive including all seven soil orders from the contiguous united states (Table 1) in which carbonates are likely to be found. This MIR model was developed dividing the dataset between calibration and test samples. Sample sizes for the calibration and validation sets are denoted by n_Cal_ and n_Val_, respectively; and **B**) a second, independent MIR prediction model based exclusively on the Cornell dataset of 209 samples collected from 5 sites in New York and Iowa and including Alfisols, Inceptisols, and Mollisols. Due to smaller total sample size (n_CV_), this calibration was performed using the cross-validation leave-one-out technique.

Models were derived at both national scale and a more regional scale to assess the effect of geographic and taxonomic coverage on the relative performance CO_3_ prediction models. The CO_3_ concentration of samples ranged from 0.00 to 494.6 g kg^-1^ with a mean and standard deviation of 1.05 and 3.18 g kg^-1^, respectively. Additionally, the organic C concentration of samples ranged from 1.5 to 662.0 g kg^-1^ with a mean and standard deviation of 14.3 and 11.4 g kg^-1^, respectively (data not shown). Excellent results were achieved generating MIR predictive models for both the KSSL national dataset and more restricted Cornell dataset (Fig 3a and 3b, respectively). R^2^ of MIR predicted versus manometrically measured values were 0.988 and 0.993 for the KSSL model and Cornell model, respectively, with negligible bias in either case. RMSEP and root mean square error of cross validation (RMSECV) were 12.7 and 7.7 g kg^-1^ for the KSSL and Cornell models, respectively. Lower error in the Cornell model is likely associated with lower chemical and spatial diversity of the dataset. While a tendency for lower error estimates from the cross validation leave-one-out method used with the smaller dataset has been reported [17] the effect of this choice was very small for this dataset. Repeating the analysis using ten randomly chosen divisions of the Cornell samples between calibration and validation subsets resulted in an average RMSEP 0.792 g kg^-1^ (range 0.639 to 0.891 g kg^-1^), essentially identical on average to the RMSECV value. RPD values of 9.0 and 11.8, respectively, suggest that both MIR models are of sufficient quality for ‘any purpose’ and not just qualitative assessment [29].

These two models, calibrated on completely independent datasets, were further tested for generality of prediction accuracy outside the original modeling datasets by evaluating the national model’s ability to accurately predict values for the independent Cornell dataset of manometrically determined CO_3_ and, conversely, the Cornell model’s ability to predict values for the much more diverse national dataset. The KSSL national model calibrated with all soil orders predicted CO_3_ contents for the independent 209 samples of the Cornell dataset (Table 2) with an RMSEP of 15.4 g kg^-1^ despite the fact that relatively few of the samples in the national dataset came from Iowa (4) or New York (18). This was a favorable result, but somewhat worse than the internal validation statistics (12.7 g kg^-1^) of the KSSL national model.

**Table 2:** Evaluating the robustness of carbonate models in reciprocal analyses.

data used for modeling^†^	Dataset analyzed	Soil order(s) analyzed	n	RMSEP	Bias	SEP^††^	RPD	Offset	Slope
**KSSL: all soil orders**									
KSSL	Cornell	all soils	209	15.4	-6.1	14.2	6.4	0.44	1.02
KSSL	Cornell	Alfisols	84	19	-9	16.7	7.3	0.84	1.01
KSSL	Cornell	Inceptisols	96	11.2	-6.4	9.1	4.2	-0.09	1.19
KSSL	Cornell	Mollisols	29	16.2	3.2	16	4.6	-0.83	1.05
**KSSL: Histosols excl.**									
KSSL	Cornell	all soils	209	10.1	-2.9	9.7	9.4	0.62	0.95
KSSL	Cornell	Alfisols	84	10.9	1.9	10.8	11.3	0.16	0.97
KSSL	Cornell	Inceptisols	96	8.1	-7.4	3.4	11.1	0.81	0.98
KSSL	Cornell	Mollisols	29	13	-1.8	13	5.7	0.74	0.94
**Cornell**									
Cornell	Cornell	all soils	209	7.2	-5 E-07	7.2	12.6	0.05	0.99
Cornell	Cornell	Alfisols	84	8.9	3 E-03	8.9	13.7	0.06	0.99
Cornell	Cornell	Inceptisols	96	3.2	0.07	3.2	11.9	-0.07	1.02
Cornell	Cornell	Mollisols	29	10.5	-0.24	10.5	7	0.39	0.96
Cornell	KSSL	all soils	1268	40.6	10	39.4	2.92	1.14	0.85
Cornell	KSSL	Alfisols	72	14.6	5.1	13.7	7.3	0.22	0.94
Cornell	KSSL	Aridisols	186	33.3	-9.8	31.9	4.35	2.44	0.91
Cornell	KSSL	Entisols	32	28.2	9.5	26.6	5.45	-0.05	0.94
Cornell	KSSL	Histosols	32	214	183	110	1.74	0.72	0.47
Cornell	KSSL	Inceptisols	70	21.4	5.6	20.7	6.02	-0.23	0.97
Cornell	KSSL	Mollisols	497	20.3	6.6	19.2	4.85	0.65	0.92
Cornell	KSSL	Vertisols	95	13.9	9.5	10.1	8.92	-0.55	0.97

The MIR CO_3_ model developed using the entire KSSL dataset and a second KSSL model excluding Histosols are both applied to the Cornell dataset. The ability of the Cornell model, in turn, to predict CO_3_ values in the KSSL dataset both in total and broken down by soil order. CO_3_ contents are in units of CaCO_3_ equivalents (see Methods) in g kg^-1^. Model evaluation statistics included are the coefficient of determination (R^2^), the root mean square of prediction (RMSEP) or RMSE of cross validation (RMSECV), bias, standard error of prediction (SEP) and the residual prediction deviation (RPD) [29].

^†^ Model parameters were: **KSSL: all soil orders**: 4002–600 cm^-1^, 1^st^ Derivative + MSC preprocessing, 13 PLS loading vectors; **KSSL but Histosols excluded**: 2982–2640, 2301–1620, 1281–939 cm^-1^. 2^nd^ Derivative preprocessing, 11 PLS factors; **Cornell**: 2301–1619 cm^-1^; 1^st^ Derivative preprocessing, 7 PLS loading vectors.

^††^ In many treatments, RMSEP and SEP are equivalent terms. In the OPUS software which generated the values shown here, they differ in that RMSEP represents the full root mean square error of prediction while SEP is bias corrected prior to calculating the root mean square. SEP is therefore lower than RMSEP. None of the models presented here had large values for overall bias, and so the differences between RMSEP and SEP presented above tend to be small.

### Influence of taxonomic coverage on model performance

**Fig 4:**
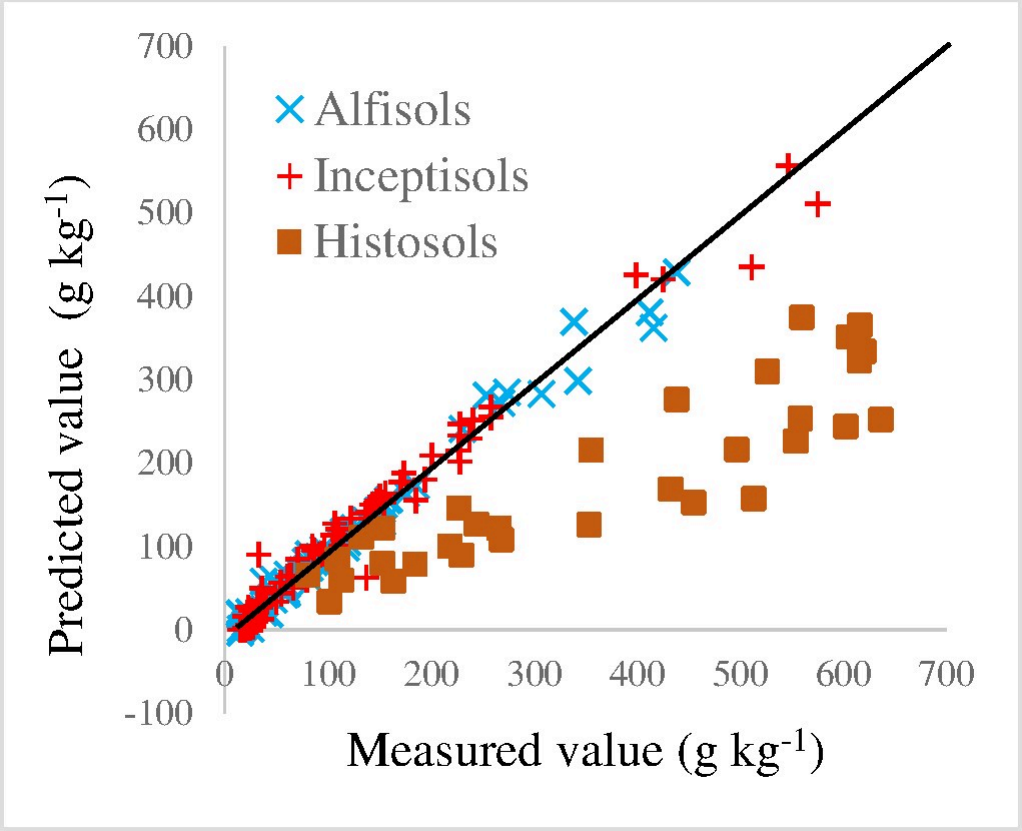
Predicted CO_3_ values using the Cornell dataset MIR calibration compared to KSSL manometrically measured CO_3_ contents for the same national database samples. Shown is the model performance for Alfisols and Inceptisols, which are the main soil orders contributing to the Cornell calibration model, and also for Histosols which are not represented in the Cornell calibration dataset at all. Statistics on these fits as well as all other soil orders present in the national dataset are given in Table 2.

When applied to the independent KSSL national dataset, the model derived from Cornell data provided moderate accuracy for Alfisols and Inceptisols from other parts of the continent with RMSEP of 14.6 and 21.4 g kg^-1^, respectively. Good performance was also seen for Vertisols with an RMSEP of 13.9 g kg^-1^ (Fig 4, Table 2) despite their absence from the calibration dataset. However, CO_3_ predictions were poor for both Aridisols and Mollisols, and were very noisy with severe underestimates for Histosols (Fig 4). Expanding the spectral region used while modeling with the Cornell dataset did not improve generality and actually worsened prediction accuracy.

An evaluation of the full KSSL national model’s (Fig 3A) internal validation data broken down by soil order is given in Table 3 along with additional calibration models for nine subset combinations of soil orders from the KSSL national dataset. In all these models, the KSSL dataset was divided into the same division of calibration and test datasets.

**Table 3:** Matrix of root mean square error of prediction (RMSEP) and bias for 10 CO_3_ prediction models all developed from the Kellog Soil Survey Laboratory (KSSL) national dataset (Table 1) and subsets thereof.

Calibration data used	Statistic	Model internal validation data	Histosols	Aridisols	Entisols	Alfisols	Inceptisols	Mollisols	Vertisols	Order unknown	Cornell data
**Full KSSL national dataset**	RMSEP	12.7	21.1	16.6	18.3	9.9	11.4	11.9	11.1	9.7	15.4
	Bias	-0.3	-6.7	0.7	6.2	0.0	0.1	0.0	-2.7	-0.4	-6.1
**w/o Histosols**	RMSEP	11.1	169.0	13.5	14.7	7.0	8.7	11.0	7.9	8.7	10.1
	Bias	0.3	132.0	-0.1	3.1	0.0	1.6	1.7	-1.0	-1.3	-2.9
**w/o Histosols or Aridisols**	RMSEP	9.8	166.0	17.1	15.5	7.1	7.6	10.7	6.4	8.4	11.1
	Bias	2.9	129.0	-4.4	3.1	1.4	1.3	1.5	-2.3	-1.8	-4.4
**w/o Histosols, Aridisols, or Entisols**	RMSEP	9.6	169.0	16.3	17.6	7.0	7.7	10.3	6.0	8.5	11.8
	Bias	0.3	133.0	0.0	5.1	0.7	0.3	1.2	-0.9	-1.8	-4.9
**Alfisols, Inceptisols & Vertisols only**	RMSEP	9.2	139.0	18.2	15.6	6.0	8.8	14.3	8.4	11.1	13.9
	Bias	0.7	96.7	-6.8	-4.1	-0.1	2.0	4.3	-0.5	3.5	-8.8
**Histosols only**	RMSEP	14.2 ^†^	N/A	83.0	59.1	85.5	61.9	84.0	124.0	88.9	77.0
	Bias	-1.8	0.6	-31.9	-34.2	-66.4	-18.9	-3.3	-105.0	-45.0	13.4
**Aridisols only**	RMSEP	12.6 ^†^	207.0	N/A	20.7	11.5	13.9	22.0	10.9	17.1	16.2
	Bias	0.1	181.0	0.0	12.0	4.1	6.0	14.3	-4.1	9.6	10.6
**Entisols only**	RMSEP	12.7 ^†^	170.0	23.3	N/A	22.6	22.6	24.7	19.9	21.4	20.5
	Bias	0.4	142.0	-6.7	0.7	-10.3	-0.4	6.0	-6.3	-4.6	0.2
**Mollisols only**	RMSEP	10.1	170.0	28.2	29.0	9.7	12.1	10.1	19.3	12.5	27.0
	Bias	0.2	121.0	-5.7	-2.1	-1.2	-2.9	0.2	-9.5	-2.5	-21.8
**w/o Histosols** 0–100 g kg^-1^ only^††^	RMSEP	5.1	ID	5.0	ID	4.6	4.0	5.4	4.5	4.5	6.3
	Bias	-0.4		-1.0		0.0	0.0	-0.4	0.2	-0.7	-4.7

All table values are in units of g kg^-1^. The data range allowed is 0–700 g kg^-1^ unless otherwise indicated. The third column shows the statistics from model validation during the calibration process. Other columns show the breakdown of model validation statistics by soil order (shaded cells in any given row) and also the ability to predict datasets not included during the calibration (unshaded cells in each row). Also shown in the right-hand column is the ability of each of the KSSL derived calibration to predict the independent Cornell dataset.

^†^ The model test data for these three single-order models are actually Root Mean Square Error of Cross Validation (RMSECV) values. Calculating a (Root Mean Square Error of Prediction (RMSEP) in these three cases would be inappropriate since it is the same as the calibration dataset, and this is indicated by N/A in the RMSEP matrix. ID indicates that there was an insufficient sample size in the restricted test set for meaningful statistics.

^††^ The calibration data and all evaluation statistics are restricted to the same 0–100 g kg^-1^ range.

Histosols, with RMSEP of 21.1 g kg^-1^, was the most poorly predicted soil order (Table 3) followed by Entisols and Aridisols with the next highest RMSEPs at 18.3 and 16.6 g kg^-1^, respectively. Excluding Histosols from model calibration resulted in an optimization using 2nd derivative preprocessing, more selective portions of the available spectrum (2982–2640, 2301–1620 and 1281–939 cm^-1^), and had a Quant2 recommended PLS using 11 factors. A substantial improvement resulted in prediction precision for all other soil orders as well as the independent Cornell dataset, but this model, like the Cornell model, was unable to make useful predictions for Histosols (Fig 4, Tables 2 and 3). This is not an important model limitation given that histosols with measureable carbonate content are extremely rare. Further excluding Entisols and Aridisols from model calibration allowed for modest additional improvement in precision for remaining orders of the KSSL dataset but less dramatically so than for Histosol exclusion, and it did not improve predictions of the independent Cornell dataset (Table 3).

Histosols represent an extreme case in which the dominant spectral background is from organic matter rather than mineral soil. Given their limited frequency and highly distinctive nature, this is not a substantive limitation to application of the restricted form of the KSSL national model. Nonetheless, inclusion of Histosols results in only modest loss of overall accuracy and may be desirable in some contexts.

Further explorations of precision for more restricted models, including calibrations based on individual soil orders, showed, as would be expected, lower RMSEP for the validation test set as the diversity of samples in the model decreased (Table 3). However, these gains were rather small, and the calibration based on all orders except Histosols actually made the best predictions of the independent Cornell dataset. More substantial improvements in accuracy were achieved by calibrating for a reduced range of CO_3_ values (Table 3), and this is recommended when high-accuracy is needed for evaluating soils at low carbonate content.

### Reproducibility and accuracy of CO_3_ measurement

Manometric assay and MIR predictions for these two KSSL laboratory CO_3_ standards were in almost perfect agreement (Table 4), and precision of individual measures was similar, possibly actually better for MIR, but the data do not include among-batch sources of error for MIR.

**Table 4:** Repeatability (within batch standard deviation (sd)), reproducibility (among batch sd) and accuracy (mean values) comparing manometric and MIR carbonate measurements done repeatedly on the same Kellogg Soil Survey Laboratory (KSSL) soil standards.

KSSL standard	Manometric CO_3_ assessment				KSSL MIR CO_3_ Prediction (calibration w/o Histosols)			
	mean	sd among batches	sd within batches	n	mean	sd among batches	sd within batches	n
	mg g^-1^	mg g^-1^	mg g^-1^		mg g^-1^	mg g^-1^	mg g^-1^	
**101**	157		0.71	14	194	5.51	4.61	2116
**104**	76.3	2.54		397	76.8		2.02	15
**146**	95.4	3.11		1228	98.0		1.52	15

**Fig 5:**
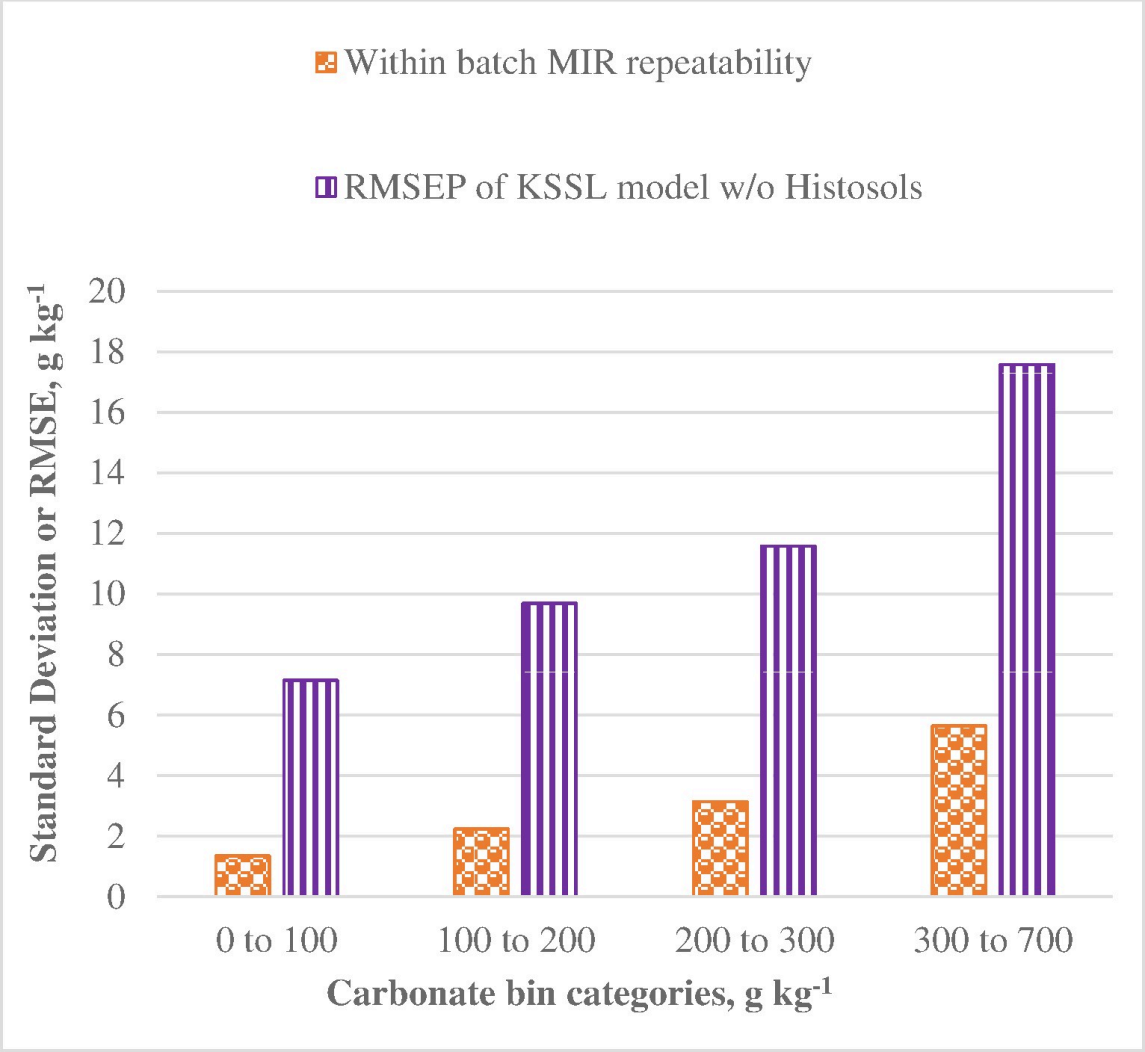
Comparison of the root mean square error of prediction (RMSEP) with the standard deviation (SD) of replicate measurements of the same soil sample within batches (96 well plates) for the KSSL national dataset without Histosols. Each reported MIR predicted value is an average of four replicate samples individually loaded and analyzed providing a robust estimate of the within-batch MIR repeatability. Repeatability was calculated as the square root of average variance in the predicted values among each set of four reps divided by square root of 4. Placement in bins was based on the manometric measurements. The number of soil samples associated with bins 0 to 100, 100 to 200, 200 to 300 and 300–700 g kg^-1^ were 300, 214, 127, and 48, respectively.

Of interest in this comparison is that, although the difference is not huge, the mean carbonate values for manometric and MIR measurement are significantly different from each other (p<0.001). Since the difference is much larger than can be explained by measurement repeatability for either assay, this suggests a spectral background effect resulting in a small but consistent overestimate of carbonate by the MIR analysis. Such a discrepancy was not seen for standards 104 or 146, but it is within the bounds of expected deviations based on the RMSEP of the KSSL national model for samples with this CO_3_ content (Fig 5).

The behavior of KSSL standard 101 is consistent with the total dataset from the KSSL national model (Fig 5). All data from the KSSL model without Histosols was divided among four CO_3_ range-based bins to allow a comparison of the reproducibility of MIR predictions for a given sample and the overall accuracy with which individual manometric measurements were captured in MIR carbonate predictions. Since each reported MIR prediction going into the model is actually an average of four reps, an expected repeatability of the measurement can be estimated from the variance. Values for within batch repeatability of MIR predictions are calculated as the square root of the average variance across all sets of four reps (independently loaded wells within a given batch) divided by the square root of four to give an expected sd for averages of four reps, the standard for reported values. Fig 5 clearly indicates that repeatability of spectral measurements of particular soil samples is only a small contributor to the overall error represented by the RMSE. This is true at all levels of CO_3_ content, but more dramatically so at low levels of CO_3_.

### Spectral regions associated with CO_3_ prediction by MIR

**Fig 6:**
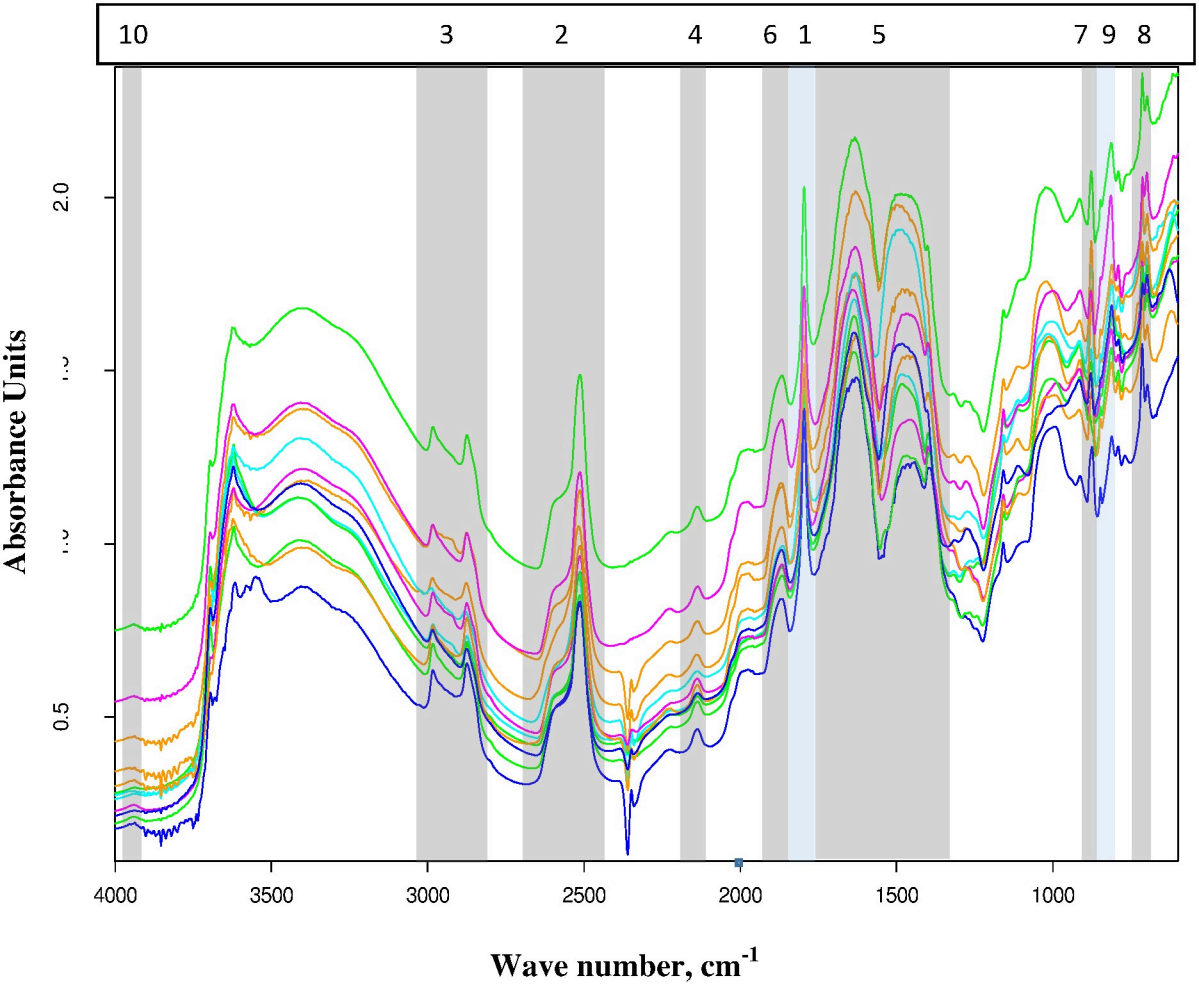
Representative spectra drawn from all soil orders in the KSSL national CO_3_ dataset except Histosols and indicating the locations of the individual bands evaluated in Table 5. All spectra shown represent soil samples with CO_3_ contents of 400–550 g kg^-1^. Contiguous bands have been given contrasting shading colors for visual clarity. Numbers at the top indicate the order in Table 3, sorted by RMSEP of predictive models based on each band individually.

To further explore the contributions of specific spectral bands to overall model performance, 10 spectral bands consistently associated with high CO_3_ were identified, and both single-band and multiband prediction models were developed (Table 5, Fig 6). Several of the listed regions have a second peak in the same interval. In some cases these may be due to variation in harmonic contributions to particular peaks, to mixed crystal structures for carbonates present in the samples, or simply closely associated separate spectral features. The crest for the most prominent peak in each spectral region is given in column 3. These bands included the expected peaks at 700, 880, and 1450 cm^-1^ [20] (699, 887, and an expected double peak in undiluted samples at 1636 and 1471 cm^-1^ in Table 5) associated with major vibrational states, a combination band 1830–1800 cm^-1^ (1796 cm^-1^ in Table 5), well-discussed overtone bands at 2600–2500 (2514 cm^-1^ in Table 5), 3000–2900 cm^-1^ [20–22] (2876 cm^-1^ in Table 5) and 3984–3937 cm^-1^ [30] (3938 cm^-1^ in Table 5), and three additional bands with consistent peaks centered at 2138, 1866 and 814 cm^-1^ (Table 5, Fig 5).

**Table 5:** MIR model results using the KSSL national dataset with Histosols excluded (n = 1101 soil samples from six soil orders) and utilizing spectral intervals corresponding to specifically identified absorbance peaks.

RMSEP rank	Spectral interval	Strongest Peak Maximum	Spread of Strongest Maximum	Secondary feature	Nature of secondary feature	RMSEP	PLS factors
#	cm^-1^	cm^-1^	cm^-1^	cm^-1^		g kg^-1^	#
1	1842–1763	1796	2	N/A	N/A	13.5	12
2	2680–2424	2514	8	2602	shoulder	23.1	13
3	3054–2826	2876	5	2985	2^nd^ Peak	29.4	9
4	2200–2103	2138	3	N/A	N/A	30.2	6
5	1758–1336	1636	13	1471	2^nd^ peak	30.6	13
6	1942–1842	1866	4	N/A	N/A	32.3	15
7	893–860	887	8	848	2^nd^ peak	34.7	13
8	745–679	699	2	714	2^nd^ peak	36	11
9	860–780	814	4	792	2^nd^ peak	45.1	12
10	3975–3930	3938	2	N/A	N/A	53.9	12

All 10 single-band models were significant, but the band from 1842–1763 cm^-1^ stands out with an RMSEP of 13.5 g kg^-1^, only 2.4 g kg^-1^ greater than the optimized national model without Histosols (Table 3). This is attributable to the strength and sharpness of this peak that make it clearly discernable against various background changes, and its greater consistency of peak centering and shape. Multiband band optimization searches using all or selected combinations of the 10 bands in Table 5 achieved RMSEP only 0.3 g kg^-1^ higher than full spectrum models (data not shown). The best multiband models always contained the peak centered at 1796 cm^-1^, but did not need all peaks and achieved similar results with a variety of ancillary band combinations. The band from 2424–2680 cm^-1^ has previously been put forward as holding the best peak for carbonate determination due to a relative lack of other soil components absorbing in that region [17,20,31]. It was the second most informative CO_3_ peak in this study, but notably less effective than the one centered at 1796 cm^-1^. The 3^rd^ and 4^th^ most informative bands (Table 5) overlap with absorbance by aliphatic compounds and carbohydrates, respectively [32].

A calibration of the KSSL national model (without Histosols) was performed using first derivative preprocessing to match the calibration analyses of individual peaks reported in Table 5. It used the full MIR spectrum available in this study (4000–600 cm^-1^), and had an RMSEP of 12.0 g kg^-1^. Spectral peaks with specific chemometric attributions are expected to show up most clearly in the first factor (loading vector) of a PLS model, but with first derivative preprocessing, the peak shapes are slightly altered [33,34]. Where a positive peak center may have been present in the raw absorbance data, the first derivative of the spectrum will have a value of 0 at this same wavelength with negative values dropping down to the left (higher wavenumbers) and positive values rising to the right reflecting the positive and negative slopes on contrasting sides of the raw absorbance maximum. The peaks ranked as the top three regarding their RMSEP (Table 5) fulfilled this expectation very well. The second and third ranked peaks from Table 5 exhibited features close to those expected in the first loading vector. These observations are consistent with the findings of previous studies, and with which peaks overlap extensively with strong absorbance by other common soil components. The expected peak at 1450 cm^-1^ for calcite splits into two rounded domes in neat samples [20]. The resulting region from 1336–1758 cm^-1^ overlaps with absorbance bands for a wide array of organic compounds [26,31]. Similarly, the expected peaks at 700 and 880 cm^-1^ overlap with absorbance by soil organic matter, quartz and clay minerals [26].

**Fig 7:**
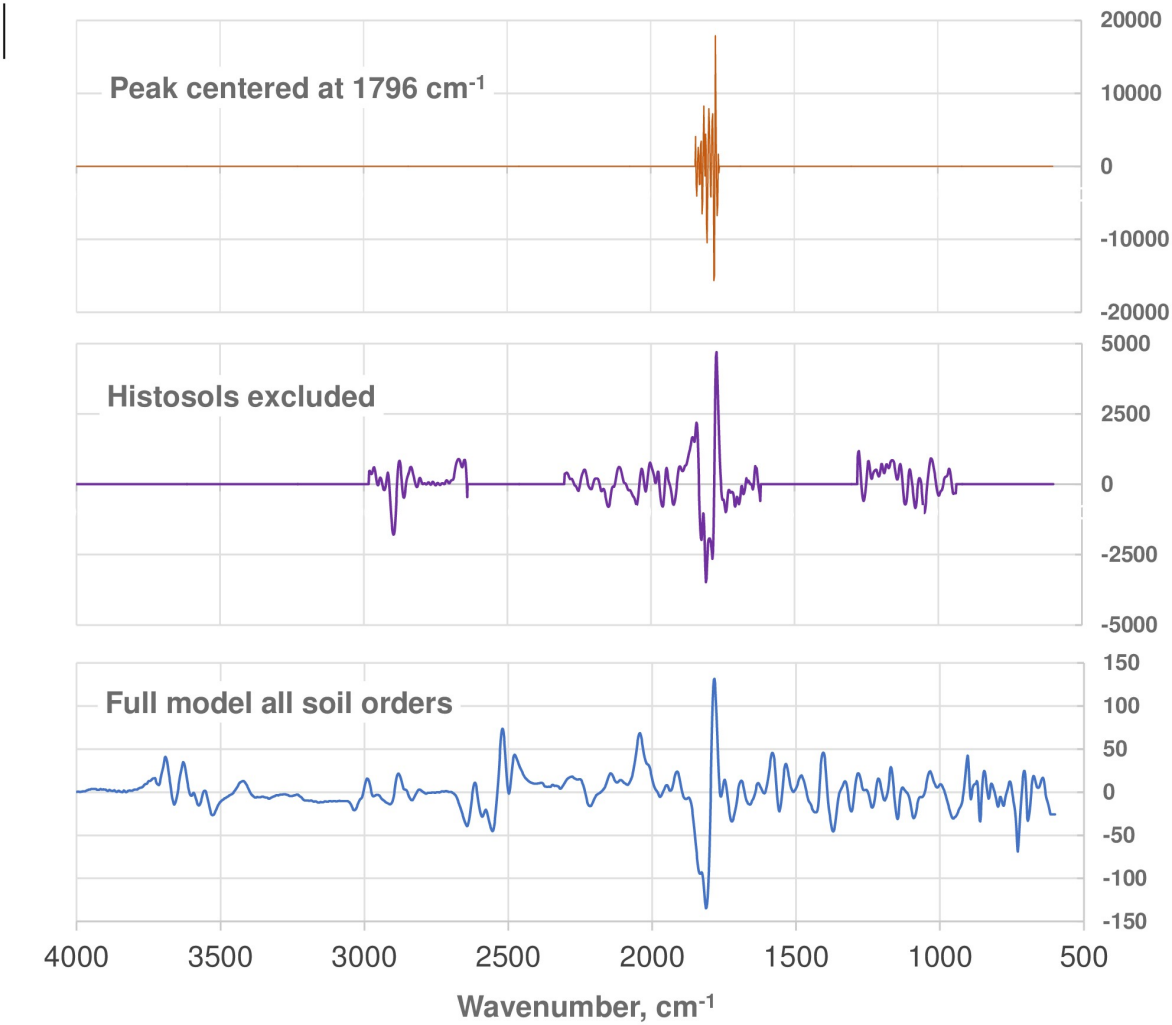
Comparison of three sets of whole model regression coefficients for CO_3_ prediction for the national dataset. **A)** PLS model using only the peak centered at 1796 cm^-1^ and 1st derivative preprocessing (Table 5). Histosols were excluded. The model used 12 loading vectors. **B)** PLS model with Histosols excluded and using 2nd derivative preprocessing (Table 3). The model used 11 loading vectors. **C)** PLS model based on all soil orders and using 1st derivative + multiplicative scattering correction preprocessing (Fig 2 & Table 3). The model had 13 loading vectors. For all three models, the full complement of final regression coefficients and all loading vectors are available as excel files in Supplemental Data online.

The PLSR regression coefficients for three of the most important CO_3_ prediction models discussed above (Peak centered at 1796 cm^-1^, Histosols excluded, and Full model all soils) are presented in Fig 7, so that the relative importance of spectral regions may be assessed. Optimization procedures resulted in the use of contrasting spectral regions for the three models, yet all emphasized the peak at 1796 cm^-1^ to a greater extent than any other region. The model based on all soil orders utilized the entire available spectrum, while optimization after exclusion of just the Histosols resulted in a substantial reduction of spectral regions utilized. The peak centered at 1796 cm^-1^ appears to be the predominant spectral region associated with CO_3_ prediction by MIR, as inclusion of additional spectral regions provided minimal gains in prediction accuracy ranging from 0.8 to 2.5 g kg^-1^.

### Advantages of MIR vs manometric assay

The MIR technique is an attractive replacement for the manometric method in several important aspects. First, MIR has a much lower propensity for human error and/or cryptic equipment failure during the procedure. At the KSSL, as much as 10% of manometric measurements are invalidated and repeated as a result of quality control procedures, and other authors have noted the difficulty of maintaining accuracy during the procedure [35]. In contrast, only a small fraction of 1% of MIR spectra ever need to be invalidated and re-collected. For the last three years, although the manometric method is still employed at the KSSL, values are always compared with prediction from MIR spectra as a powerful QC procedure. Discrepancies greater than +/- 2 times the RMSEP of the MIR model are tagged for a re-analysis by the manometric measurement. The KSSL lab currently uses the national MIR model for QA/QC of data generated by the manometric method.

The MIR spectral approach also has lower cost once the initial investment in spectrometers has been made. At the KSSL, processing, archiving and entering the soil into the database are in themselves a fairly costly procedure. The additional cost of performing a manometric CO_3_ measurement versus MIR spectral analysis is then $3.60 versus $1.80 per sample, respectively. The lower cost for the MIR analysis is a result of higher throughput, reduced labor, and with no chemical costs. Further, of course, the same spectra can be used for numerous other predictions of other soil properties, and if the costs of multiple lab bench assays were considered the savings would become even more dramatic.

While spectral windows exist where CO_3_ peaks suffer little overlap with other key soil parameters, the reverse may be less true and the many strong carbonate peaks are likely to interfere with many other measurement goals. The effectiveness with which carbonates can be predicted, however, may provide opportunities for MIR model trees [36] and stratification.

This study demonstrates the value of a large, diverse, well-studied and well-curated soil collection and database in generating high-performance MIR models. The KSSL soil archive and associated characterization database provides unique opportunities to derive high-quality MIR models for a wide range of soil properties. Access to the spectral library is freely available upon request.

## Conclusions

After investment in the instrumentation, spectroscopic data can be generated with low cost per sample and high throughput. They have the potential advantage of providing information about numerous soil properties at once if a set of high-quality models are available for interpretation. While benchtop analytic methods at their best may have somewhat higher precision than full-range MIR prediction models, specialized models with comparable precision can be developed where needed. The low frequency of spectral measurement error compared to chemical methods can also result in greater overall accuracy unless very extensive QA/QC procedures are in place.

Even with Histosols included, CO_3_ MIR models encompassed geographically and chemically diverse soils with minimal bias and excellent precision, suitable for most analytical needs. This validates Hypothesis 1 and has been fully demonstrated here for soils of the continental United States. The localized Cornell model was very limited outside its calibration dataset and error increased even with soils of the same order from other regions of the continent. In contrast, the national KSSL models proved to be robust. This confirms Hypothesis 2, and suggests broader, global models will be quite possible, but maintaining optimal precision and accuracy will require adding new soil orders into the calibration dataset.

In a strict sense, Hypothesis 3 was rejected for this dataset. The MIR prediction models were strongly affected by spectral background and this uncertainty was the primary contribution to RMSEP, which were larger than repeatability measures for either MIR or manometric assay. In terms of percent error, this was particularly true for samples with low carbonate contents. Nonetheless, very good predictions were possible and, in terms of full scale of the range of CO_3_ contents, the differences between manometric and MIR uncertainty were small. For many contexts, the MIR models are suitable as primary analytical assays.

The peak at 1796 cm^-1^ exhibited the least evidence of confounding overlap with other soil properties and gave excellent results even as a single peak prediction model. The peak at 2514 cm^-1^ was also quite good, but with an RMSEP nearly twice that of the peak at 1796 cm^-1^.
